# Did the Relative Age Effect Change Over a Decade in Elite Youth Ski Racing?

**DOI:** 10.3389/fspor.2019.00055

**Published:** 2019-11-05

**Authors:** Lisa Steidl-Müller, Erich Müller, Carolin Hildebrandt, Christian Raschner

**Affiliations:** ^1^Department of Sport Science, University of Innsbruck, Innsbruck, Austria; ^2^Department of Sport Science and Kinesiology, University of Salzburg, Salzburg, Austria

**Keywords:** ski racing, talent development, relative age quarter distribution, discrimination, selection error

## Abstract

The relative age effect (RAE) represents an asymmetry in birth quarter distribution, favoring athletes born early in the selection year and discriminating against late born athletes. The RAE was proven to be present in all age categories of national and international levels of alpine ski racing. Due to the existence of the RAE in all categories, it can be assumed that a selection error takes place favoring early born and early maturing youth ski racers. However, whether selection strategies have changed during the last years due to the high amount of research done in this field, has not been investigated so far in this sport. Therefore, the aim of the present study was to assess whether the magnitude of the RAE in youth ski racers aged 10–14 years has changed during the last decade by comparing the periods 2005–2009 (“former” athletes) and 2015–2019 (“current” athletes). Pupils of a well-known skiing-specific secondary modern school as well as members of the provincial ski team, who all competed at national levels, were included in the study. Next to the birth months, anthropometric characteristics (body height, weight, body mass index) were assessed. Chi-square tests were used to compare differences between the observed and expected relative age quarter distributions across five age categories (U11–U15). Additionally, Kruskal–Wallis-*H*-Tests were performed to assess differences in anthropometric characteristics between athletes of the four relative age quarters. Mann–Whitney *U*-Tests were performed to assess possible differences in anthropometric characteristics between former and current ski racers. A highly significant RAE was present in both former [χ^2^_(3, 764)_ = 60.36; *p* < 0.001; ω = 0.31] and current youth ski racers [χ^2^_(3, 702)_ = 43.13; *p* < 0.001; ω = 0.29] with an over-representation of athletes of Q1 (30.3–34.2%) and a clear under-representation of athletes of Q4 (14.8–15.0%). Generally, results indicated no change in the magnitude of the RAE in youth alpine ski racing over the past 10–15 years, emphasizing the robust nature of this phenomenon. No significant differences were found in any of the anthropometric characteristics between athletes of the four relative age quarters in both former and current athletes, indicating that relatively younger athletes of the last relative age quarter seem to have to have advanced anthropometric characteristics for being selected. Changes in the talent selection process should be performed to reduce the impact of the RAE.

## Introduction

The *relative age effect* (RAE) represents a well-documented phenomenon in youth sports context, and was first documented in Canadian ice hockey (Barnsley et al., 1985). It exists when the relative age quarter distribution of selected athletes represents a biased distribution with an over-representation of athletes born early in the selection year, meaning close to the cut-off date for the competition categories (Musch and Grondin, 2001). Even though the intention of grouping children and youth athletes into competition categories based on their chronological age is to guaranteeing fair competition and reflecting age-related development, age differences of up to 12 months are possible between athletes competing in the same category. These relative age advantages are defined as the RAE; its presence has been proven in several different types of sport, such as soccer (Helsen et al., 2005; Cobley et al., 2009; Romann and Fuchslocher, 2013), ice hockey (Hurley et al., 2001), basketball (Delorme and Raspaud, 2009), volleyball (Nakata and Sakamoto, 2013), baseball (Nakata and Sakamoto, 2012), swimming (Medic et al., 2009), tennis (Edgar and O'Donoghue, 2005), among others. Talent selection systems are often based on selection biases that confuse maturation for talent (Baker et al., 2014). In this context, Baker et al. (2014) proposed the *maturation-hypothesis* for explaining the RAE, assuming that the relative age of an athlete is related to the athlete's cognitive and physical maturation. Thus, maturational differences between relatively older and relatively younger athletes seem to influence the favorable selection of the relatively older ones (Baker et al., 2014). As a short-term consequence, relatively older and more mature athletes seem to be potentially more “talented,” which leads to the favorable selection of them, whereas relatively younger and less mature athletes are often over-looked and do not get the same changes for fulfilling their potential (Malina et al., 2004; Romann and Cobley, 2015). Knowing that talent in a sport does not depend on the birth month, the existence of the RAE indicates that the talent development systems in these sports are biased and that many young talented athletes are discriminated against (Lames et al., 2008; Cobley et al., 2009).

The RAE is present also in alpine ski racing at both national and international levels. It starts to exist at the youngest age groups of national levels with athletes aged 7–11 years (Müller et al., 2015a), continuing on national levels with athletes aged up to 14 and 15 years (Romann and Fuchslocher, 2014; Müller et al., 2015a). At international youth and adolescent levels, the RAE is present among ski racers participating at the International Children's Games (12–15 years; Müller et al., 2017b), the European Youth Olympic Festival (17–18 years; Müller et al., 2016a), the Youth Olympic Games (15–16 years; Raschner et al., 2012), as well as the Junior World Ski Championships (16–20 years; Müller et al., 2012). At elite level, the RAE is also present among World Cup athletes (Müller et al., 2012; Baker et al., 2014; Bjerke et al., 2016). At most age categories and levels, the RAE exists among both male and female athletes (Steidl-Müller et al., 2019). The likelihood for selection for national final races among 10–12 year old youth ski racers is up to 3.4 times higher for an athlete born in the first 3 months of the year compared with an athlete born between July and September and even 5.1 times higher compared with an athlete born in the last 3 months (Müller et al., 2017a). The biological maturation additionally influences the selection process in youth ski racing because relatively younger athletes, meaning athletes born late in the selection year, seem to “only” have a chance for selection for national final races if they are early maturing. More than 40% of the selected ski racers for the national final races born in the last quarter were early maturing, whereas in an age-matched comparison group of non-athletes a normal distribution of early, normal, and late maturing pupils was found in each quarter (Müller et al., 2017a). Additionally, hardly any late maturing ski racers were found in the cohort of selected athletes for national final races in general independent of their relative age quarter (Müller et al., 2017a). Thus, selection processes in youth alpine ski racing are influenced by the relative age and the biological maturation of an athlete; a fact which leads to the necessity of changes in the talent development systems in order to guarantee more fairness for all athletes independent of their relative age and biological maturity status (Steidl-Müller et al., 2019).

Research has focused on the RAE and the influential mechanisms of it during the last years. Several solutions were proposed to minimize the effect, such as rotating cut-off dates (Hurley et al., 2001), changing cut-off dates (Cobley et al., 2009), implementing corrective adjustments (Romann and Cobley, 2015) etc. However, thus far, the effectiveness of these suggestions has not been proven (Romann and Fuchslocher, 2014). Whether the extent of the RAE has changed during the last years after the research focus on it in several types of sport was investigated only in European soccer. Helsen et al. (2012) examined whether 10 years of research has made any difference in the RAE in professional soccer in ten European countries and did not find any changes in the occurrence and strength of the RAE after 10 years (2000/2001 season vs. 2010/2011 season) indicating the robust nature of this phenomenon. However, whether the extent of the RAE in alpine ski racing has changed after the first research studies in 2012 (Müller et al., 2012) and later on, has not been investigated, so far.

Ski boarding schools play an important role in the talent development in ski racing in Austria. The schools concentrate on the dual career of the athletes combining school and sport. Next to the schools, the provincial ski teams represent a serious cornerstone in the development of young talents in this sport (Raschner et al., 2015). The social significance of alpine ski racing in Austria is reflected in the high number of young athletes starting with this sport already at the age of 5–6 years; up to 200 athletes per birth year compete in children's races at this age. Based on these numbers, not surprisingly, a high selection pressure is present in this sport in Austria and the first selection already takes place as early as the age of 9–10 years, when the entrance exams for ski boarding schools are held, as well as at the age of 12–13 years for the teenager squad of the provincial ski teams (Raschner et al., 2015). The awareness of the RAE problem has increased during the last years among coaches of the boarding schools and the provincial ski teams, but it is not clear whether the research output had also affected the selection processes in this sport. Therefore, the aim of the present study was to assess whether the magnitude of the RAE in youth ski racers aged 10–14 years has changed during the last decade by comparing the periods 2005–2009 (“former” athletes) and 2015–2019 (“current” athletes). It was hypothesized that the magnitude of the RAE may have diminished during the last decade due to the greater awareness of the consequence of this selection bias.

## Materials and Methods

### Participants

In the present study, elite youth ski racers, who all competed in ski races at national level, were included. They were pupils of well-known Austrian skiing-specific secondary modern schools or members of the provincial ski team. The ski racers can be divided into two groups: 764 participants (428 male, 336 female) tested from 2005 to 2009, and 702 participants (390 male, 312 female) tested 10 years later (2015–2019). The athletes ranged in age between 9.6 and 14.5 years (mean age: 12.5 ± 1.2 years). To minimize effects of one single season, each 5 consecutive years were summarized per age group (U11–U15) and subsequently defined as “former” (2005–2009) and “current” (2015–2019) period. Table 1 presents the number of participants per age group divided by the two 5-year periods (2005–2009 vs. 2015–2019) and per gender.

**Table 1:** Number (*n*) of participants per age group divided by former (2005–2009) and current (2015–2019) period.

**Sample**	**Age category**	**2005–2009**	**2015–2019**
Total sample	U11	68	36
	U12	140	151
	U13	178	235
	U14	201	116
	U15	177	164
Males	U11	38	23
	U12	83	76
	U13	94	130
	U14	111	67
	U15	102	94
Females	U11	30	13
	U12	57	75
	U13	84	105
	U14	90	49
	U15	75	70

### Procedures

The procedures are in accordance with the ethical standards of the Declaration of Helsinki and were approved by the Institutional Review Board. The birth dates of all participants were collected prior to the assessment of the anthropometric characteristics. The birth months were then categorized into four relative age quarters. In alpine ski racing in Austria, the cut-off date for the competition categories is January 1. Therefore, the birth months were split into quarters to calculate the relative age quarters as follows: January to March was categorized as relative age quarter 1 (Q1); April to June as quarter 2 (Q2); July to September as quarter 3 (Q3), and October to December as quarter 4 (Q4).

The anthropometric characteristics were assessed at the end of the winter season in April or May of the single calendar year. The body height (0.5 cm) was recorded using a portable stadiometer SECA 217 (Seca, Hamburg, Germany). Body mass (1 N) was measured on a Kistler force plate (Kistler Instrumente AG, Gommiswald, Switzerland) with normal sports clothes but without shoes, and was then calculated to the nearest 0.1 kg (divided by 9.81). The body mass index (BMI; 0.1 kg/m^2^) was then calculated as body mass in kilograms divided by height in meter squared (Nuttall, 2015).

### Statistical Analyses

To assess the difference between the observed and the expected relative age quarter distributions, chi^2^-tests (χ^2^) were used for the former (2005–2009) and current (2015–2019) youth ski racers. The relative age quarter distribution of the Austrian population of the same birth years as the participants, which corresponded to a nearly even distribution among the four quarters (nearly 25% in each quarter), was used as the expected distribution for these analyses. The effect size ω was calculated for the χ^2^-tests (Sherar et al., 2007). Odds ratio (OR) and 95% confidence intervals (95% CI) were calculated (Cobley et al., 2009). Additionally, χ^2^-tests and the corresponding Cramer's *V* were also used to assess differences in the birth quarter distributions between the former (2005–2009) and current (2015–2019) youth ski racers over all age categories, as well as for each age group separately. All analyses were performed for the total sample of the two 5-year periods as well as separated by gender. In order to assess possible differences in the anthropometric characteristics between the four relative age quarters, Kruskal–Wallis-*H*-Tests were performed (due to small sample sizes in each category) for each age category as well as separated by the two 5-year periods. Additionally, possible differences in the anthropometric characteristics between former and current athletes were assessed using Mann–Whitney *U*-Tests separated by relative age quarter, age category, and gender.

The level of significance was set at *p* < 0.05. All of the calculations were performed using IBM SPSS 25.0 (IBM Corporation, Armonk, NY, USA); the effect size ω was assessed using G^*^Power 3.1.9.2 (University of Düsseldorf, Germany).

## Results

### Presence of the Relative Age Effect

**Figure 1:**
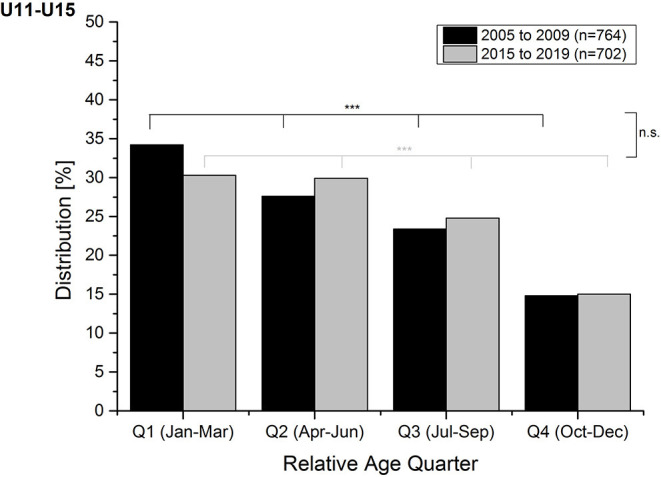
Relative age quarter distributions of former (2005–2009; black) and current (2015–2019; light gray) ski racers of all age groups (U11–U15). ^***^p < 0.001; n.s., not significant difference between groups.

A highly significant RAE was found for both former [χ^2^_(3, 764)_ = 60.36; *p* < 0.001; ω = 0.31) and current youth ski racers [χ^2^_(3, 702)_ = 43.13; *p* < 0.001; ω = 0.29] with an over-representation of athletes of Q1 and a clear under-representation of athletes of Q4 (14.8–15.0%). The distribution of the male athletes of both groups significantly differed from the expected distribution as well [former: χ^2^_(3, 428)_ = 34.22; *p* < 0.001; ω = 0.32; current: χ^2^_(3, 390)_ = 34.57; *p* < 0.001; ω = 0.39] with larger effect sizes in the current group. The relative age quarter distribution of the female athletes significantly differed from the expected distribution in both groups, as well [former: χ^2^_(3, 336)_ = 26.36; *p* < 0.001; ω = 0.31; current: χ^2^_(3, 312)_ = 13.10; *p* = 0.004; ω = 0.21] with larger effect sizes in the former group of athletes. The relative age quarter distributions of the total sample of former (2005–2009) and current (2015–2019) athletes are presented in Figure 1. Table 2 presents the distributions of both 5-year periods for the different age groups and separated by gender. The descriptive OR and the corresponding χ^2^ for each quarter of the total sample and both male and female athletes of the former and the current ski racer groups are presented in Table 3. With respect to the former ski racers (2005–2009), the OR calculations revealed significant differences between Q1 and all other quarters in the total sample as well as between Q1 and Q3 and Q4 in the male athletes and between Q1 and Q4 in the female athletes. The calculations revealed additionally, that among the current ski racers (2015–2019), differences were found between Q1 and Q3 and Q4 in the total sample, as well as between Q1 and Q4 in both male and female athletes and between Q1 and Q3 in female athletes. The likelihood of selection for the ski boarding school or the provincial ski teams was up to 2.50 times higher for an athlete of Q1 compared with an athlete of Q4 in the current group and up to 2.35 times higher in the former group.

**Table 2:** Relative age quarter distributions of male and female former (2005–2009) and current (2015–2019) youth ski racers.

		**Period**	***n***	**Q1 [%]**	**Q2 [%]**	**Q3 [%]**	**Q4 [%]**	**Diff. 2005–2009 vs. 2015–2019**		
								**χ^2^**	***p***	**Cramer's *V***
Total sample	Total	2005–2009	764	34.2	27.6	23.4	14.8	2.61	0.456	0.04
		2015–2019	702	30.3	29.9	24.8	15.0			
	U11	2005–2009	68	20.6	30.9	33.8	14.7	4.21	0.239	0.20
		2015–2019	36	8.3	47.2	27.8	16.7			
	U12	2005–2009	140	34.3	27.9	26.4	11.4	8.73	**0.033**	0.17
		2015–2019	151	28.5	26.5	20.5	24.5			
	U13	2005–2009	178	32.6	27.5	23.6	16.3	7.17	0.067	0.13
		2015–2019	235	36.2	32.8	23.0	8.1			
	U14	2005–2009	201	34.8	28.4	19.9	16.9	3.15	0.369	0.10
		2015–2019	116	31.0	24.1	28.4	16.4			
	U15	2005–2009	177	40.1	25.4	20.9	13.6	5.93	0.115	0.13
		2015–2019	164	28.0	29.3	28.0	14.6			
Males	Total	2005–2009	428	34.1	27.3	24.1	14.5	2.61	0.456	0.06
		2015–2019	390	30.8	29.7	27.2	12.3			
	U11	2005–2009	38	23.7	42.1	34.2	0	9.85	**0.020**	0.40
		2015–2019	23	13.0	43.5	21.7	21.7			
	U12	2005–2009	83	36.1	27.7	22.9	13.3	2.06	0.561	0.11
		2015–2019	76	26.3	30.3	25.0	18.4			
	U13	2005–2009	94	30.9	25.5	24.5	19.1	12.75	**0.005**	0.24
		2015–2019	130	40.8	30.8	23.8	4.6			
	U14	2005–2009	111	35.1	24.3	21.6	18.9	4.53	0.210	0.16
		2015–2019	67	28.4	22.4	35.8	13.4			
	U15	2005–2009	102	38.2	26.5	23.5	11.8	3.09	0.378	0.13
		2015–2019	94	26.6	29.8	28.7	14.9			
Females	Total	2005–2009	336	34.2	28.0	22.6	15.2	2.22	0.528	0.06
		2015–2019	312	29.8	30.1	21.8	18.3			
	U11	2005–2009	30	16.7	16.7	33.3	33.3	9.06	**0.029**	0.46
		2015–2019	13	0	53.8	38.5	7.7			
	U12	2005–2009	57	31.6	28.1	31.6	8.8	11.17	**0.011**	0.29
		2015–2019	75	30.7	22.7	16.0	30.7			
	U13	2005–2009	84	34.5	29.8	22.6	13.1	0.69	0.875	0.06
		2015–2019	105	30.5	35.2	21.9	12.4			
	U14	2005–2009	90	34.4	33.3	17.8	14.4	1.16	0.762	0.09
		2015–2019	49	34.7	26.5	18.4	20.4			
	U15	2005–2009	75	42.7	24.0	17.3	16.0	3.53	0.317	0.16
		2015–2019	70	30.0	28.6	27.1	14.3			

*Q, relative age quarter; Q1, January–March; Q2, April–June; Q3, July–September; Q4, October–December. Bold values highlight significant differences in the relative age quarter distributions between former and current youth ski racers*.

**Table 3:** Descriptive odds ratio across all relative age quarters of former (2005–2009) and current (2015–2019) youth ski racers.

**Sample**		**Q1:Q2**		**Q1:Q3**		**Q1:Q4**	
		**2005–2009**	**2015-2019**	**2005-2009**	**2015-2019**	**2005–2009**	**2015–2019**
Total sample	Chi^2^	5.30	0.02	15.28	3.93	58.57	36.68
	*p* value	0.021	0.884	<0.001	0.047	<0.001	<0.001
	OR [95% CI]	**1.24 (1.09–1.41)**	1.02 (0.88–1.15)	**1.46 (1.27–1.67)**	**1.22 (1.06–1.41)**	**2.31 (1.95–2.73)**	**2.03 (1.70–2.42)**
Male athletes	Chi^2^	3.20	0.07	7.43	0.87	33.92	30.86
	*p* value	0.074	0.795	0.006	0.352	<0.001	<0.001
	OR [95% CI]	**1.25 (1.05–1.48)**	1.03 (0.86–1.24)	**1.42 (1.18–1.70)**	1.31 (0.94–1.36)	**2.35 (1.88–2.95)**	**2.50 (1.93–3.23)**
Female athletes	Chi^2^	2.11	0.00	0.87	3.88	24.68	8.64
	*p* value	0.146	0.942	0.352	0.049	<0.001	0.003
	OR [95% CI]	**1.22 (1.01–1.48)**	0.99 (0.81–1.21)	**1.51 (1.23–1.87)**	**1.37 (1.09–1.71)**	**2.25 (1.76–2.90)**	**1.63 (1.28–2.07)**

*OR, odds ratio; CI, Confidence Interval; Q1–4, relative age quarter 1–4. Bolded values indicate significance of odds ratio (if 95% CI does not include 1)*.

### Changes in the Relative Age Effect Over the Decade

**Figure 2:**
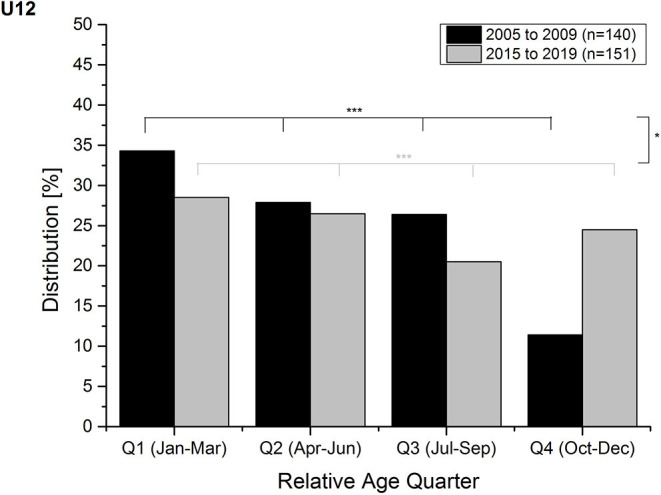
Changes in the relative age quarter distributions of former (black) to current (light gray) U12 youth ski racers. ^***^p < 0.001; ^*^p < 0.05.

The relative age distribution of the current ski racer group did not significantly differ from the distribution of the former group of athletes neither in the total sample nor in male or female athletes (see Table 2). When separating by age group, a significant difference was found in the U12 age group of the total sample (see Figure 2), showing a stronger RAE in the former athletes (*V* = 0.17). Among male athletes, significant differences between relative age quarter distributions of the former and the current youth ski racers were evident in the U11 and U13 age groups with a stronger RAE in the former U11 athletes (*V* = 0.40) and a stronger RAE in current U13 ski racers (*V* = *0*.24). Similar results were found among female athletes: significant differences in the relative age quarter distributions were present in U11 (stronger RAE of current athletes; *V* = 0.46) and U12 (stronger RAE among former athletes; *V* = 0.29).

### Differences in Anthropometric Characteristics Between Relative Age Quarters

The anthropometric characteristics of former (2005–2009) and current (2015–2019) youth ski racers of the four relative age quarters are presented separated by age group for male athletes in Table 4 and for female athletes in Table 5. No significant differences were found in any of the three anthropometric characteristics as well as in any age group between the athletes of the four relative age quarters neither in the former athletes nor in the current athletes. Significant differences between former and current male ski racers were found in body height in the U12 age group of athletes born in Q2 (*z* = −2.222; *p* = 0.026), in body weight in U14 athletes of Q1 (*z* = −2.038; *p* = 0.042) and in BMI in U14 (*z* = −2.659; *p* = 0.008) and U15 athletes of Q1 (*z* = −2.092; *p* = 0.036). In female athletes significant differences between former and current athletes were found in body weight in U12 athletes of Q4 (*z* = −2.552; *p* = 0.011) and in BMI in U12 athletes of Q4 (*z* = −2.130; *p* = 0.033) and in U13 athletes of Q2 (*z* = −2.059; *p* = 0.039).

**Table 4:** Anthropometric characteristics (M ± SD) and inferential statistics of former (2005–2009) and current (2015–2019) male youth ski racers divided by relative age quarter.

		**Q1 (Jan-Mar)**	**Q2 (Apr-June)**	**Q3 (July-Sept)**	**Q4 (Oct-Dec)**	**Kruskal–Wallis H**	***p***
**Body height [cm]**							
U11	2005–2009 (*n* = 38)	140.2 ± 5.5	142.6 ± 6.2	143.5 ± 4.8	x	1.33	0.515
	2015–2019 (*n* = 23)	147.7 ± 6.0	140.6 ± 5.9	142.2 ± 4.8	145.9 ± 6.9	4.54	0.209
U12	2005–2009 (*n* = 83)	146.8 ± 6.2	148.9 ± 7.1	146.7 ± 5.9	146.6 ± 9.1	1.95	0.582
	2015–2019 (*n* = 76)	146.5 ± 4.9	144.8 ± 4.9	145.5 ± 5.0	148.9 ± 5.0	5.62	0.132
U13	2005–2009 (*n* = 94)	153.3 ± 7.6	152.6 ± 6.0	151.7 ± 6.8	150.6 ± 7.5	1.54	0.673
	2015–2019 (*n* = 130)	152.8 ± 5.4	151.0 ± 6.0	150.4 ± 5.8	149.7 ± 7.7	5.15	0.161
U14	2005–2009 (*n* = 111)	159.6 ± 8.3	157.5 ± 7.3	157.4 ± 9.2	155.4 ± 8.0	3.53	0.317
	2015–2019 (*n* = 67)	157.6 ± 7.1	158.3 ± 6.8	159.0 ± 8.8	156.6 ± 3.6	0.35	0.950
U15	2005–2009 (*n* = 102)	166.0 ± 8.3	166.3 ± 9.2	166.3 ± 9.0	163.4 ± 10.3	0.70	0.873
	2015–2019 (*n* = 94)	166.0 ± 8.0	166.5 ± 7.1	166.5 ± 8.1	168.9 ± 8.6	0.81	0.846
**Body weight [kg]**							
U11	2005–2009 (*n* = 38)	34.8 ± 4.2	34.6 ± 6.0	37.2 ± 6.0	x	1.17	0.556
	2015–2019 (*n* = 23)	38.5 ± 8.8	32.5 ± 3.9	35.3 ± 5.8	39.7 ± 7.3	3.83	0.281
U12	2005–2009 (*n* = 83)	37.6 ± 5.9	40.6 ± 7.5	40.3 ± 7.2	38.6 ± 8.6	2.26	0.520
	2015–2019 (*n* = 76)	37.6 ± 5.6	36.5 ± 4.0	37.8 ± 5.1	38.5 ± 3.5	2.32	0.509
U13	2005–2009 (*n* = 94)	43.0 ± 6.3	42.3 ± 6.3	43.0 ± 7.9	41.6 ± 7.3	1.22	0.748
	2015–2019 (*n* = 130)	42.2 ± 4.8	41.0 ± 5.7	42.7 ± 7.5	39.3 ± 5.1	1.92	0.589
U14	2005–2009 (*n* = 111)	49.1 ± 8.2	46.5 ± 6.4	47.6 ± 8.4	46.1 ± 7.6	2.91	0.405
	2015–2019 (*n* = 67)	44.3 ± 5.9	45.1 ± 7.3	50.6 ± 10.9	44.5 ± 5.0	3.85	0.278
U15	2005–2009 (*n* = 102)	55.4 ± 7.6	56.6 ± 9.4	54.9 ± 9.4	52.7 ± 9.9	1.91	0.590
	2015–2019 (*n* = 94)	53.2 ± 7.8	54.7 ± 9.3	56.9 ± 9.9	55.0 ± 9.1	1.22	0.749
Body mass index [kg*m^**−2**^**]**							
U11	2005–2009 (*n* = 38)	18.1 ± 1.4	16.9 ± 2.4	18.1 ± 2.2	x	4.30	0.116
	2015–2019 (*n* = 23)	17.5 ± 3.7	16.4 ± 1.2	17.4 ± 2.1	18.5 ± 1.8	3.92	0.270
U12	2005–2009 (*n* = 83)	17.6 ± 1.7	18.0 ± 2.4	18.7 ± 2.4	17.7 ± 2.0	2.46	0.483
	2015–2019 (*n* = 76)	17.5 ± 2.1	17.3 ± 1.5	17.8 ± 2.0	17.4 ± 1.1	0.97	0.808
U13	2005–2009 (*n* = 94)	18.3 ± 1.8	18.1 ± 2.0	18.7 ± 2.4	18.2 ± 1.7	0.62	0.893
	2015–2019 (*n* = 130)	18.1 ± 1.8	17.9 ± 1.7	18.8 ± 2.3	17.6 ± 0.9	4.04	0.257
U14	2005–2009 (*n* = 111)	19.2 ± 1.7	18.7 ± 1.5	19.2 ± 2.1	19.0 ± 1.6	1.12	0.773
	2015–2019 (*n* = 67)	17.8 ± 1.5	18.0 ± 1.9	19.9 ± 2.7	18.1 ± 1.6	7.51	0.057
U15	2005–2009 (*n* = 102)	20.0 ± 1.7	20.3 ± 1.7	19.8 ± 1.9	19.7 ± 2.1	2.18	0.537
	2015–2019 (*n* = 94)	19.3 ± 1.9	19.6 ± 2.1	20.4 ± 2.4	19.2 ± 1.8	4.92	0.178

**Table 5:** Anthropometric characteristics (M ± SD) and inferential statistics of former (2005–2009) and current (2015–2019) female youth ski racers divided by relative age quarter.

		**Q1 (Jan-Mar)**	**Q2 (Apr-June)**	**Q3 (July-Sept)**	**Q4 (Oct-Dec)**	**Kruskal–Wallis H**	***p***
**Body height [cm]**							
U11	2005-2009 (*n* = 30)	145.2 ± 4.0	142.8 ± 4.6	139.6 ± 7.2	139.7 ± 5.1	6.87	0.076
	2015–2019 (*n* = 13)	x	142.2 ± 4.6	140.8 ± 3.7	144.0	0.72	0.697
U12	2005–2009 (*n* = 57)	145.6 ± 8.2	145.5 ± 6.3	144.8 ± 7.4	143.5 ± 2.9	2.24	0.525
	2015–2019 (*n* = 75)	146.3 ± 6.0	145.3 ± 4.1	146.2 ± 5.5	148.4 ± 7.5	2.98	0.395
U13	2005–2009 (*n* = 84)	152.6 ± 7.7	152.9 ± 7.6	149.1 ± 5.2	152.4 ± 8.6	5.22	0.156
	2015–2019 (*n* = 105)	154.1 ± 6.3	152.0 ± 5.9	150.6 ± 6.2	152.3 ± 6.4	3.85	0.278
U14	2005–2009 (*n* = 90)	157.5 ± 7.3	158.6 ± 5.9	155.8 ± 5.5	160.2 ± 6.1	3.32	0.345
	2015–2019 (*n* = 49)	160.9 ± 6.6	159.6 ± 4.4	158.7 ± 7.7	158.3 ± 7.8	1.04	0.792
U15	2005–2009 (*n* = 75)	161.4 ± 5.1	163.0 ± 5.7	162.3 ± 5.8	165.3 ± 5.1	4.89	0.180
	2015–2019 (*n* = 70)	163.5 ± 5.5	164.1 ± 4.1	163.0 ± 6.8	161.0 ± 4.9	2.78	0.426
**Body weight [kg]**							
U11	2005–2009 (*n* = 30)	33.8 ± 3.0	37.3 ± 4.5	32.5 ± 4.6	31.5 ± 1.7	3.59	0.309
	2015–2019 (*n* = 13)	x	33.7 ± 3.0	32.3 ± 4.0	37.4	1.79	0.409
U12	2005–2009 (*n* = 57)	38.9 ± 7.2	37.6 ± 5.3	35.1 ± 5.1	34.1 ± 2.4	4.67	0.197
	2015–2019 (*n* = 75)	37.0 ± 4.5	36.3 ± 4.6	36.0 ± 5.4	39.8 ± 5.0	7.25	0.064
U13	2005–2009 (*n* = 84)	43.9 ± 7.2	44.5 ± 7.8	39.2 ± 4.4	43.0 ± 8.1	7.33	0.062
	2015–2019 (*n* = 105)	43.4 ± 6.1	41.3 ± 6.4	41.3 ± 5.4	43.0 ± 6.3	2.71	0.438
U14	2005–2009 (*n* = 90)	49.9 ± 8.5	49.7 ± 7.6	45.1 ± 4.6	51.3 ± 6.9	6.72	0.081
	2015–2019 (*n* = 49)	51.0 ± 7.8	49.5 ± 7.1	45.6 ± 4.1	50.7 ± 9.1	2.57	0.464
U15	2005–2009 (*n* = 75)	53.4 ± 7.3	54.9 ± 5.9	51.5 ± 4.8	56.3 ± 7.4	4.23	0.240
	2015–2019 (*n* = 70)	56.1 ± 8.0	55.2 ± 5.7	55.2 ± 7.3	55.9 ± 9.7	0.15	0.985
Body mass index [kg*m^**−2**^**]**							
U11	2005–2009 (*n* = 30)	16.3 ± 0.0	18.0 ± 1.5	16.6 ± 1.5	16.5 ± 1.0	2.76	0.431
	2015–2019 (*n* = 13)	x	16.6 ± 1.0	16.2 ± 1.2	18.0	2.33	0.312
U12	2005–2009 (*n* = 57)	17.8 ± 1.6	17.6 ± 1.6	16.8 ± 1.4	16.6 ± 1.0	6.00	0.112
	2015–2019 (*n* = 75)	17.3 ± 1.5	17.2 ± 1.6	16.8 ± 1.6	18.1 ± 1.4	7.31	0.063
U13	2005–2009 (*n* = 84)	18.8 ± 1.9	18.9 ± 1.9	17.6 ± 1.4	18.5 ± 2.4	5.09	0.166
	2015–2019 (*n* = 105)	18.2 ± 1.7	17.8 ± 1.9	18.2 ± 1.4	18.5 ± 1.9	2.97	0.396
U14	2005–2009 (*n* = 90)	20.1 ± 2.6	19.7 ± 2.3	18.6 ± 1.4	20.1 ± 2.2	4.85	0.183
	2015–2019 (*n* = 49)	19.7 ± 2.4	19.4 ± 2.4	18.4 ± 1.5	20.1 ± 2.2	2.95	0.399
U15	2005–2009 (*n* = 75)	20.5 ± 2.5	20.7 ± 2.0	19.6 ± 1.8	20.6 ± 2.6	2.57	0.462
	2015–2019 (*n* = 70)	21.0 ± 2.6	20.5 ± 2.0	20.8 ± 2.2	21.5 ± 2.7	1.28	0.733

## Discussion

The present study was the first study that assessed the longitudinal development of the magnitude of the RAE in youth alpine ski racing. In both groups of selected athletes, former and current ski racers, and among both males and females, a highly significant RAE was found. No significant change in the magnitude of the RAE was evident when comparing former and current youth ski racers in total or divided by gender and age category. Only in U12 athletes, a weaker RAE was assessed among current athletes.

Due to the importance of the two cornerstones in the talent development in youth ski racing (ski boarding schools and provincial ski teams), selected athletes of both organizations were included in the present study. Due to the high selection pressure, not surprisingly, a significant RAE was found in male and female former and current youth ski racers, which is in line with several studies assessing the RAE in youth alpine ski racing (Steidl-Müller et al., 2019). There has been neither a decrease nor an increase in the prevalence of the RAE over a decade (*p* > 0.05). The relative age quarter distribution showed that 30.3% of the current athletes were born in the first quarter and only 15.0% were born in the last quarter, compared with 34.2 and 14.8%, respectively in former athletes. Additionally, similar effect sizes were calculated for both groups (former: ω = 0.31; current: ω = 0.29). When differentiating by gender, similar results were found showing no significant change in the prevalence of the RAE in both males and females (*p* > 0.05). However, in male athletes a stronger effect size was evident in the current athletes (ω = 0.39 vs. ω = 0.32), whereas among the female ski racers a weaker effect size was calculated in the current youth ski racer group (ω = 0.21 vs. ω = 0.31). However, in both males and females an over-representation of athletes of Q1 and a clear under-representation of athletes of Q4 was evident. These results are in line with the comparison of the prevalence of the RAE between 2000/2001 and 2010/2011 elite European soccer players, in which no significant change was found (Helsen et al., 2012). Despite the great research interest and the proposed solutions to minimize the RAE, there has been little impact on the effect (Helsen et al., 2012). Only in the U12 age category a significant decrease in the prevalence of the RAE was found in the total sample of male and female ski racers over the decade. However, the fact that in the next age group (U13) an obviously stronger RAE was present again in the current group compared with the former U13 group, but also compared with the U12 current group, leads to the speculation that the selection for the provincial ski teams, which takes place at the age of approximately 12 years, might clearly favor relatively older athletes during the last 5 years and therefore, might strengthen again the RAE. Though, when comparing the distributions of the male and female current and former athletes separately, different trends are apparent. A significant weaker RAE was found in male U11 athletes of the current group (*V* = 0.40), whereas in the females a stronger RAE was present in the current group (*V* = 0.46). However, the small sample sizes have to be considered in the U11 age groups (males: 38 vs. 23; females: 30 vs. 13). Conversely, in female U12 athletes a weaker RAE was present in the current group (*V* = 0.29), whereas in male U13 athletes a stronger RAE was evident in the current group (*V* = 0.24). One might speculate that nowadays young girls enter puberty even earlier than it might have been a decade ago, which could probably explain why the RAE, which is much influenced by the maturation-based selection, was stronger in the current U11 group, but was weaker in the current U12 group. In the study performed by Helsen et al. (2012), elite soccer players were investigated, thus, no age categories were considered in the analyses, which does not allow any comparisons, though.

The most frequent explanation for the RAE phenomenon is the maturation-selection hypothesis (Baker et al., 2014), which was confirmed also in youth alpine ski racing showing that the most valuable influential factor of the RAE seems to be the biological maturity status (Steidl-Müller et al., 2019). Relatively younger athletes seem to “only” have a chance for selection if they are early maturing, which was confirmed by the findings that 43% of athletes born in Q4 who were selected for national final races (11–12 years) were early maturing (Müller et al., 2017a), a finding which was observed also in youth soccer (Deprez et al., 2013) and basketball (Torres-Unda et al., 2013), as well. Additionally, youth ski racers of the last relative age quarter that were selected for national final races significantly differed in biological maturity status from Q4-athletes that were not selected in both males and females (Müller et al., 2016b). In alpine ski racing an advanced biological maturity status is advantageous in the selection process because more mature athletes benefit from early recognition from coaches and talent scouts (Müller et al., 2017a). Based on this assumption, relatively younger athletes can counteract their relative age disadvantage by an advanced maturation and thus, it can be assumed that they represent similar or more advanced anthropometric characteristics compared with the athletes of the other relative age quarters. The biological maturity status was not measured in the present study, because the implementation of the prediction of the age at peak height velocity (APHV; Mirwald et al., 2002) in the regular fitness testing of youth ski racers was conducted starting in 2015 onwards; however, the prediction of the APHV is based on anthropometric measurements (Mirwald et al., 2002) and anthropometric characteristics such as body height and weight correlate with the biological maturation. Thus, in the present study anthropometric characteristics were compared between athletes of the four relative age quarters in both former and current ski racers. In contrast to the study of Müller et al. (2015b), in which youth ski racers born in Q1 had significantly higher values in body height and weight, no significant differences were found in anthropometric characteristics between the four relative age quarters in both former and current ski racers. Thus, it can be assumed that the ski racers of the four relative age quarters were comparable with respect to their maturation, a finding which would be in line with previous studies including the measurement of the biological maturation and showing no differences between athletes of the four relative age quarters (Müller et al., 2015b, 2016b, 2017a). Similar results were found also in studies in soccer, in which no differences in the biological maturity status between athletes of the four relative age quarters were evident (Gil et al., 2014; Lovell et al., 2015). As a consequence, it can be speculated that relatively younger athletes of the fourth relative age quarter are more mature when they have the same anthropometric characteristics as athletes who are some months older. Even though it only can be speculated because the biological maturity status was not measured, it might underline the supposed necessity of advanced anthropometric characteristics of relatively younger ski racers for getting the possibility of being selected irrespective of their relative age disadvantage. And due to the performance advantages of advanced anthropometric characteristics in alpine ski racing (Raschner et al., 1995), it can be hypothesized that this could partly explain why both former and current ski racers of the four relative age quarters did not differ in anthropometric characteristics between each other. In general, former and current male and female ski racers did not significantly differ in anthropometric characteristics without differentiating between quarters. Only among athletes of single quarters and age categories differences were found. However, whether for example the higher body mass in current female athletes of Q4 in the U12 age group has led to the reduced prevalence of the RAE in the current group can only be speculated. Nevertheless, all these results emphasize the favorable selection of relatively older and more mature athletes in ski racing and this has not changed during the last decade.

However, it has to be considered that the selection of the participants for this study was performed in that way that all youth ski racers who were either pupils of the skiing-specific boarding schools or members of the provincial ski team in the given periods were included. This has to be seen as limitation of the study, because the study did not include all athletes that competed at national levels in this period. Another limitation of the study is that one athlete could have been included in several age categories, which might represent a bias. However, the analyses were performed for each age category separately, and the information if an athlete drops out of sport at a given time point and is not again included in the next age category seems also important.

## Conclusion

The magnitude of the RAE in youth alpine ski racing has not changed over the last 10–15 years. Despite the great research interest in this field and the proposed solutions to minimize the RAE, there has been little impact on the effect, as it was also the case in soccer (Helsen et al., 2012). Several suggestions to reduce the RAE, such as corrective adjustments or delaying the selection process of talent identification beyond stages of puberty and maturation (Cobley et al., 2009), do not seem applicable in alpine ski racing due to the changing outdoor environment that does not provide the possibility of calculating fair corrective adjustments as it is possible in swimming, or the early-specialization character of this sport (Steidl-Müller et al., 2019). As proposed in a recent review article (Steidl-Müller et al., 2019), changes in the competition system, as for example introducing a rotating cut-off date, seem to possibly be most effective for reducing the RAE. In addition, even though the awareness of the RAE problem has risen among coaches in youth ski racing during the last years, it does not seem to have changed selection criteria. As a consequence, the extent and the consequences of the RAE and the favorable selection of early born and early maturing ski racers should be spread even more during the education of coaches working with youth athletes. The long-term consequences of the selection error leading to the RAE should become more evident in the entire talent development system in this sport in order to get the chance to minimize the discrimination of relatively younger and late maturing athletes.

## Data Availability Statement

All datasets generated for this study are included in the article.

## Ethics Statement

The studies involving human participants were reviewed and approved by Institutional Review Board of the Department of Sport Science of the University of Innsbruck. Written informed consent to participate in this study was provided by the participants' legal guardian/next of kin.

## Author Contributions

LS-M, CH, and CR collected the data. LS-M analyzed the data, prepared tables and figures, and wrote the manuscript. EM analyzed and interpreted the data. All authors designed the study, contributed to data interpretation and manuscript revision, and read and approved the submitted version.

### Conflict of Interest

The authors declare that the research was conducted in the absence of any commercial or financial relationships that could be construed as a potential conflict of interest. The handling editor declared a past supervisory role and past co-authorships with one of the authors EM.
